# Hypoglycemic effect of the aqueous extract from *Acalypha argomuelleri* Briq. 'Sweet stick' leaves in *Rattus rattus* var. *albinus*

**DOI:** 10.12688/f1000research.164673.1

**Published:** 2025-08-19

**Authors:** Jorge Guillermo Morales Ramos, Berta Loja Herrera, Néstor Manuel Rodríguez Alayo, Doyle Isabel Benel Fernández, Luis Felipe Pérez Medina, Carolina Susana Loayza Estrada, María Ema Soledad Mocarro Willis, César Sánchez Marín

**Affiliations:** 1 Faculty of Human Medicine, Universidad De San Martín de Porres, Lambayeque, Peru; 2 Faculty of Human Medicine, Universidad Mayor de San Marcos, Lima, Peru; 3 Faculty of Human Medicine, Universidad Nacional Pedro Ruiz Gallo, Lambayeque, Lambayeque, Peru; 4 Program of Human Medicine, Universidad Señor de Sipán, Lambayeque, Peru; 5 Research Directorate, Hospital Regional Lambayeque, Lambayeque, Peru

**Keywords:** Diabetes mellitus, Hypoglycemic effect, aqueous extract, Acalypha argomuelleri Briq

## Abstract

**Introduction:**

Diabetes mellitus (DM) is a chronic metabolic disease representing a global public health concern and is associated with severe complications such as cardiovascular and renal diseases. Although several species of the genus *Acalypha* have demonstrated biological activity, no prior studies have evaluated the hypoglycemic effect of *Acalypha argomuelleri* Briq., making this study relevant.

**Method:**

The hypoglycemic effect of the aqueous leaf extract of *A. argomuelleri* Briq. (AAAE) was evaluated in an experimental model using *Rattus r*attus var. albinus. A randomized, prospective design was employed, consisting of a control group and three treatment groups receiving doses of 100, 150, and 300 mg/kg of the extract, respectively. Hyperglycemia was induced via oral glucose administration.

**Results:**

The qualitative phytochemical analysis of AAAE revealed the presence of flavonoids, phenols, cardiotonic glycosides, and diterpenes, with no reducing sugars. The 300 mg/kg dose produced a significant and sustained reduction in blood glucose levels, reaching near-normal values at 90 minutes, demonstrating a dose- and time-dependent hypoglycemic effect.

**Discussion:**

The study confirmed that AAAE has a dose-dependent hypoglycemic effect, with optimal efficacy at 300 mg/kg. This dose showed a faster and more sustained reduction in glucose levels compared to 100 and 150 mg/kg, suggesting higher efficacy at elevated concentrations. The identified flavonoids and phenols, associated with glucose metabolism modulation and pancreatic β-cell protection, likely explain the observed effect. The absence of reducing sugars indicates the hypoglycemic effect is linked to secondary metabolites.

**Conclusions:**

The AAAE exhibited a significant dose- and time-dependent hypoglycemic effect, with optimal efficacy at 300 mg/kg after 90 minutes. These findings support the potential of *A. argomuelleri* Briq. as a natural alternative for blood glucose control, though further studies are needed to assess its safety and efficacy in clinical models.

## 1. Introduction

The World Health Organization (WHO) defines Diabetes mellitus (DM) as a chronic metabolic disease characterized by elevated blood glucose levels. DM causes complications such as vascular diseases, heart conditions, blindness, renal failure, and neurological disorders. Type 1 diabetes (T1D), also known as insulin-dependent diabetes, is a chronic condition where the pancreas produces little or no insulin, which cannot be prevented. In contrast, Type 2 diabetes (T2D) typically occurs in adults, is the most common form, and is frequently associated with obesity. T2D can be prevented by avoiding complications and premature death. ^1^

In 2022, an estimated 828 million adults worldwide had DM, with prevalence increasing in 155 countries for men and 132 for women. Between 1990 and 2022, age-standardized prevalence rose from 7% to 14%, justifying the need to explore alternative treatments. ^2^

In the Americas, 112 million adults over the age of 18 are living with DM. According to data released in a report by the Pan American Health Organization (PAHO), under the heading ‘country profiles - diabetes disease burden, 2023’, revealed that DM (excluding diabetic kidney disease) increased and that the total number of deaths in 2019 was 141 812 distributed in middle-to-high income groups. ^3^

A projection for 2045 in Latin America and the Caribbean (LAC) estimated that the number of people with DM would reach 49 million, with a prevalence of 11.9%. Globally, it is projected that by 2035, DM2 cases would rise to 643 million, a 50% increase from current figures. ^4^ A review study concluded that individuals with T2DM in Latin American developing countries face a higher risk of cardiovascular or all-cause mortality compared to those in developed countries. ^5^

Conventional therapies for DM1 include insulin administration, while DM2 relies on oral hypoglycemic agents such as metformin. The American Diabetes Association (ADA) 2024 guidelines recommend GLP-1 analogs and SGLT2 inhibitors as first-line treatments for MN2 patients with cardiovascular disease (CV), heart failure, or chronic kidney disease. ^6^

Hypoglycemia has been exposed as one of the most serious adverse side effects of anti-diabetic treatments. Numerous epidemiological studies have highlighted the importance of a diet rich in plant foods including vegetables, fruits, spices and condiments in the prevention and treatment of DM. Various plants are used for their anti-diabetic and hypoglycemiant c effects, such as *Morus alba* L. (‘mulberry’), *Juglans regia* L. (‘walnut’), which are widely used throughout the world. In Peru, there is a wide diversity of plant species with hypoglycemiant effects, some of which belong to traditional medicine and are being studied for their use and toxicity, as in the case of *Geranium ayavacense* L. (‘pasuchaca’). ^7^

Various parts of the plant have been used to obtain extracts and identify their metabolites. Among the widely studied antidiabetic plants, *M. alba* “mulberry” stands out, whose metabolites present in the leaves include flavonoids such as rutin and quercetin-3-triglyceride, which were shown to have hypoglycemic and pharmacological effects on DM2 in animal models and humans, as synthesized in our systematic review. ^8– 11^

Another plant to consider is *A. wilkesiana* Müll. Arg. whose aqueous extract prepared from its leaves revealed the presence of bioactive principles such as: coumarins, polyphenols, flavins, terpenes, tannins and saponosides, which have a potentiating effect on diabetic nephropathy. ^12^

In Iran, the hydrosol of *J. regia* is traditionally used to regulate blood sugar in patients with DM. Various bioactive metabolites have been identified in it, including flavonoids, phenolic compounds, limonene, myrcenes, linalool, beta-pharmesene, borneol monoterpenes, caffeic acid and coumaric acids, sinapic acid, ferulic acid, juglone, 1,4-naphthoquinone, 3-3′-bisjuglone, cyclo-tri-juglone, regiolone, tripertenes, betulin and botulinic acid. ^13– 16^

In Peru, several medicinal plants, such as *Geranium ayavacense* Linnaeus. and *Geranium ruizii* Hieron (‘pasuchaca’), are recognized in traditional medicine for their medicinal properties. These plants have demonstrated hypoglycemic effects in experimental models using hyperglycemic rats ( *Rattus rattus* var. *albinus*), with leaves are used in infusions and flowers in hydroethanolic extracts. Other species with similar therapeutic properties include *Psoralea glandulosa* Linnaeus. (‘cullen’), *Physalis angulata* Linnaeus (‘mullaca’), and *Smallanthus sonchifolius* (Poepp.) H. Rob (‘yacón’). ^17– 20^

Numerous studies on the genus *Acalypha* have demonstrated its pharmacological properties, including antimicrobial effects ( *A. integrifolia* and *A. wilkesiana*), anti-inflammatory effects ( *A. fruticosa* Forsskal.), and anticancer effects ( *A. monostachya*). ^21– 24^

*Acalypha argomuelleri* Briq., commonly known as ‘Sweet stick’, is a species distributed from Ecuador to Peru. It is a shrub that primarily grows in the humid tropical biome. Taxonomically, it belongs to the class Equisetopsida, subclass Magnoliidae, order Malpighiales, family Euphorbiaceae, and the genus *Acalypha.* Euphorbiaceae is one of the largest plant families, comprising numerous genera and approximately 6,300 species; Acalypha is the third largest genus within this family, including around 500 species. ^25, 26^

This study aimed to assess the hypoglycemic effect of the aqueous extract of *Acalypha argomuelleri* Briq. (EAAA) using an experimental model, as no scientific evidence is currently available regarding its medicinal properties. Despite its traditional use in folk medicine, the therapeutic potential of this species remains unexplored in the scientific literature. Therefore, this research seeks not only to address this gap but also to investigate the potential of *Acalypha. argomuelleri* as a natural alternative for glycemic control—an approach that could prove beneficial for communities that rely heavily on traditional medicine.

## 2. Methods

The study was carried out between January and December 2024. During this period, the following stages were completed: collection and botanical identification of the plant material, conditioning and acclimatisation of the experimental animals, as well as the preparation and administration of the alcoholic extract, followed by that of the aqueous extract of Acalypha argomuelleri Briq. The experimental phase was then carried out in the experimental physiology laboratory of the Universidad Nacional Pedro Ruiz Gallo between June and December. Blood glucose measurements were taken at specific times according to the experimental design, and the data obtained were statistically analysed to evaluate the hypoglycaemic effect of the extract.

The research followed a quantitative, true experimental approach, appropriate for the study as it allows the establishment of causal relationships between the administration of the extract and changes in glucose levels. The study was prospective with a longitudinal design. A completely randomized design with control and treatment groups was used to evaluate the hypoglycemic effect of the aqueous extract of *A. argomuelleri* (AEAA) in hyperglycemic *Rattus rattus* var. albinus.

a. The rats were housed in metal cages containing sterile wood shavings. The temperature was controlled at 20–24 °C, the humidity at 50–60%, and the lighting was on a 12-hour cycle. They were fed a standard diet and had access to water at all times. No environmental enrichment was applied.
  a. The protocol avoided painful procedures. Nontoxic doses were used, and animals were monitored regularly.
  b. There were no adverse events.
  c. Humane endpoints were not established for the study as no serious harm was expected. Animals were monitored twice daily.

The corresponding permits were obtained from the National Forest and Wildlife Service (SERFOR) (Resolutions No. D000120-2023-MIDAGRI-SERFOR-ATFFS-CAJAMARCA and No. D000167-2024) for the collection of *Acalypha argomuelleri* Briq., as well as authorization from the Research Department of the Lambayeque Regional Hospital (HRL), including approval from the Institutional Research Ethics Committee for the use of animals (No. 026-2025).

The collection of *A. argomuelleri* Briq. leaves at the flowering stage were carried out in the district of Querocoto, Chota province, Peru. In situ plant samples were taken and transported to the National University of Trujillo, where they were deposited in the Herbarium Truxillensis (Trujillo–La Libertad) for proper identification and registration.

No previous protocol was registered en a public database. The experimental design was reviewed and approved by the Hospital ethics Committee.

### Preparation of the aqueous extract from *Acalypha argomuelleri* Briq. leaves

For the preparation of the aqueous extract of A. argomuelleri (AEAA), the method described by García-Granados ^27^ was used, which involved the following steps:

1. Drying: The leaves were cleaned and dried at room temperature until a constant weight was achieved.
2. Grinding: The dried leaves were ground until they reached an approximate size of 2 mm.
3. Extraction: 200 g of plant material were placed in 300 mL of water at 100°C for 45 minutes. Subsequently, the mixture was filtered, pressed, and reduced to a final volume of 162 mL.
4. Concentration: The liquid was dried in an oven at 42°C for 48 hours, followed by cooling in a desiccator. By weight difference, a dry residue of 16.5 g was obtained.
5. Preparation of Solutions: Based on the dry extract, 25 mL solutions were prepared at concentrations of 100, 150, and 300 mg/mL, respectively.

### Phytochemical analysis

A qualitative phytochemical analysis of the aqueous extract of *Acalypha argomuelleri* Briq. was carried out using the drop assay, following the method described by Dueñas-Deyá. ^28^ The presence of metabolites was determined based on specific colorimetric reactions for each group of compounds. The intensity of the color reaction qualitatively indicated the presence or absence of bioactive components using the following scale:

- Absent (–)
- Present in small amount (+)
- Present in moderate amount (++)
- Present in abundance (+++)

To indicate the presence of sugars, the Benedict’s test was used following the methodology described by García-Granados. ^27^

### Experimental design

The study was conducted with 33 adult rats ( *Rattus rattus* var. *albinus*), aged between 4 and 6 months, weighing between 190 and 230 gr., and divided into four experimental groups. The control group initially consisted of 9 rats, while each of the three treatment groups included 8 rats. To ensure sample size homogeneity and to meet the assumptions of Analysis of Variance (ANOVA), the statistical analysis was performed considering 8 rats per group over a period of six months.

A hyperglycemic state was induced in the rats through oral administration of glucose (5 mL). Subsequently, the aqueous extract of *Acalypha argomuelleri* Briq. was administered orally in three different doses (100 mg/kg, 150 mg/kg, and 300 mg/kg). Blood glucose levels were recorded under preprandial conditions, first determining the basal level and then the hyperglycemic level, with measurements taken at 30, 60, and 90 minutes after extract administration. Blood glucose was measured using an ACCU-CHEK glucometer and test strips, selected for their accuracy and reliability, as supported by previous studies.

No additional environmental enrichment was applied, which is a recognized but common limitation in preclinical pharmacological studies.

### Statistical analysis

Data analysis was performed using SPSS software version 22.0, applying single and double-entry statistical tests. To compare the groups, an Analysis of Variance (ANOVA) based on the “F” distribution was used for multiple comparisons, followed by Duncan’s post hoc test. Results with a p-value < 0.05 were considered statistically significant.

There was no blinding at any stage of the experiment.

## 3. Results

### 3.1 Phytochemical profile of the aqueous extract of *Acalypha argomuelleri* Briq.

The qualitative phytochemical analysis revealed the presence of several bioactive compounds. The results are summarized in the following table:

**Table:** 

Constituent	Test performed	Presence in the leaf
Diterpenes	Cooper acetate test	++
Cardiotonic glycosides	Keller-Kilani test	+
Phenols	Ferric chloride test (12.5%)	+++
Flavonoids	Shinoda test	++

The qualitative phytochemical analysis revealed an abundant presence of phenols, a moderate amount of flavonoids and diterpenes, and a small quantity of cardiotonic glycosides. Reducing sugars were not detected. These findings suggest that the flavonoids and phenols present in the extract may be linked to the observed hypoglycemic effect, given their potential to modulate enzymatic activity and protect pancreatic β-cells.

### 3.2 Blood glucose in the control group

The blood glucose data from the control group rats (0 mL/kg), which did not receive treatment with AEAA, served as the reference baseline to compare the effects observed in the treated experimental groups. Basal glucose levels and the progressive reduction in blood glucose levels over time (30, 60, and 90 minutes) were monitored. These measurements showed variation among individuals, reflecting natural fluctuations in glucose levels.

The data observed in Table 2 showed the glucose response in rats treated with AEAA at a dose of 100 mg/kg. Basal glucose measurements ranged from 99 mg/dL to 124 mg/dL, with an average of 111 mg/dL, which were found to be within the normal range. The highest recorded hyperglycemia peak was 342 mg/dL. At 30 minutes, most rats exhibited a significant increase in blood glucose levels. By 60 minutes, a variable decrease was observed, with some rats maintaining elevated levels, and at 90 minutes, a progressive reduction occurred, though some rats retained high levels.

**Table 1.** Glucose response in rats treated with AEAA (Control).

Rat	Weight (g)	Extract dose (mL/kg)	Basal glucose (mg/dL)	Glucose 30 min (mg/dL)	Glucose 60 min (mg/dL)	Glucose 90 min (mg/dL)
1	200	0	132	200	194	186
2	216	0	104	196	141	229
3	200	0	101	200	213	198
5	172	0	96	186	185	63
6	215	0	89	181	190	105
7	200	0	112	183	213	198
8	220	0	140	200	170	181
9	170	0	100	211	197	190

**Table 2.** Glucose response in rats treated with AEAA (100 mg/kg).

*Acalypha argomuelleri* (100 mg/kg)							
Rat	Weight (g)	Extract dose (mL/kg)	Basal glucose (mg/dL)	Hyperglycemia	Glucose 30 min (mg/dL)	Glucose 60 min (mg/dL)	Glucose 90 min (mg/dL)
1	329	0.140	121	342	156	179	180
2	268	0.110	116	155	286	256	219
3	295	0.120	124	289	131	129	130
4	286	0.120	99	256	300	152	127
5	298	0.130	115	279	201	121	99
6	266	0.110	115	160	281	250	160
7	326	0.140	120	332	178	166	120
8	286	0.120	101	256	300	152	110

At this dose, AEAA appears to induce a significant hyperglycemia peak at 30 minutes in some rats, suggesting a variable response. However, glucose levels did not decrease uniformly among all rats, as some maintained elevated levels up to 90 minutes. Although the response to the 100 mg/kg dose was not entirely uniform it appears to indicate that AEAA may have a glucose-regulating effect, which acted which acted following an initial hyperglycemic peak, with some rats showing a gradual reduction in their glucose levels over time.

The results shown correspond to rats treated with AEAA at a dose of 150 mg/kg. The average basal glucose was found to be 101 mg/dL, and the hyperglycemia peak reached a maximum of 220 mg/dL. Regarding glucose progression, a less pronounced increase was observed at 30 minutes compared to the 100 mg/kg dose. By 60 minutes, a more uniform decrease was seen in most rats, and at 90 minutes, greater stability in values with a tendency toward normalization was noted. This suggests that, given the more consistent glucose reduction at 60 and 90 minutes and fewer individual fluctuations, the extract at this dose may better regulate glucose absorption, potentially making it more effective at stabilizing glucose levels ( Table 3).

**Table 3.** Glucose response in rats treated with AEAA (150 mg/kg).

*Acalypha argomuelleri* (150 mg/kg)							
Rat	Weight (g)	Extract dose (mL/kg)	Basal glucose (mg/dL)	Hyperglycemia	Glucose 30 min (mg/dL)	Glucose 60 min (mg/dL)	Glucose 90 min (mg/dL)
1	385	0.288	95	206	188	165	166
2	306	0.229	102	184	158	142	148
3	287	0.215	108	162	145	134	126
4	328	0.246	96	220	158	140	118
5	288	0.216	110	188	166	145	136
6	384	0.288	106	192	169	145	133
7	285	0.215	105	194	168	133	112
8	304	0.220	100	180	147	130	105

In summary, the data from the third treatment with AEAA at a dose of 300 mg/kg indicated an average basal glucose of 78 mg/dL, while hyperglycemia peak reached a maximum of 391 mg/dL. Regarding glucose progression, extreme variability was obserbed at 30 minutes, with some rats showing high values. By 60 minutes, a more controlled decrease was observed. At 90 minutes, glucose levels were lower compared to the other doses. The interpretation, given de lower glucose levels observed, suggested that 300 mg/kg of AEAA might be the most effective dose for sustained glucose reduction and could have a long-term regulatory effect ( Table 4).

**Table 4.** Glucose response in rats treated with AEAA (300 mg/kg).

*Acalypha argomuelleri* (300 mg/kg)							
Rat	Weight (g)	Extract dose (mL/kg)	Basal glucose (mg/dL)	Hyperglycemia	Glucose 30 min (mg/dL)	Glucose 60 min (mg/dL)	Glucose 90 min (mg/dL)
1	200	0.240	70	143	93	93	94
2	180	0.216	82	391	170	158	151
3	190	0.228	72	146	149	146	138
4	200	0.240	86	148	133	139	114
5	180	0.216	80	284	363	123	97
6	210	0.252	55	135	140	101	92
7	170	0.204	92	161	117	110	113
8	187	0.224	76	163	151	155	123

Table 5, analyzed the results using the arithmetic mean ( $\bar{x}$ ), standard deviation (σ), and coefficient of variation (CV), with the following interpretations:

**Table 5.** General Interpretation of the Mean ( $\bar{x}$ ), Standard Deviation (σ), and Coefficient of Variation (CV%) in glucose response in rats treated with AEAA.

Etapa	Weight (gr)	Basal (mg/dL)	Hyperglycemia (mg/dL) 2.5 ml glucose	30 min	60 min	90 min	N
** Control (Without Extract)**	$\bar{x}$ =196.09 σ=20.07 CV=10.24	$\bar{x}$ =104.89 σ=21.23 CV=20.24	$\bar{x}$ =196.78 σ=13.97 CV=7.10	$\bar{x}$ =214.33 σ=77.70 CV=36.25	$\bar{x}$ =186.89 σ=73.56 CV=39.36	$\bar{x}$ =156.56 σ=43.66 CV=27.89	9
**Treatment 1 AEAA 100 mg/kg**	$\bar{x}$ =294.25 σ=23.46 CV=7.97	$\bar{x}$ =113.88 σ=9.14 CV=8.03	$\bar{x}$ =258.63 σ=69.85 CV=27.01	$\bar{x}$ =229.13 σ=70.05 CV=30.57	$\bar{x}$ =175.63 σ=51.23 CV=29.17	$\bar{x}$ =143.13 σ=40.38 CV=28.91	8
**Treatment 2 AEAA 150 mg/kg**	$\bar{x}$ =320.88 σ=41.69 CV=12.99	$\bar{x}$ =102.75 σ=5.47 CV=5.32	$\bar{x}$ =190.75 σ=17.30 CV=9.07	$\bar{x}$ =162.38 σ=13.74 CV=8.46	$\bar{x}$ =141.75 σ=10.95 CV=7.73	$\bar{x}$ =130.50 σ=19.90 CV=15.25	8
**Treatment 3 AEAA 300 mg/kg**	$\bar{x}$ =189.63 σ=13.14 CV=6.93	$\bar{x}$ =76.63 σ=11.33 CV=14.78	$\bar{x}$ =196.38 σ=92.13 CV=46.92	$\bar{x}$ =164.50 σ=83.51 CV=50.77	$\bar{x}$ =128.13 σ=25.01 CV=19.52	$\bar{x}$ =115.25 σ=21.34 CV=18.52	8

Low CV (<10%): Homogeneous data, stable response.

Moderate CV (10%–30%): Acceptable variability.

High CV (>30%): High dispersion, unstable response.

For glucose variation without AEAA application (Day 0), a normal response curve was observed following glucose administration, showing an initial high peak (214.33 mg/dL) and a progressive reduction as metabolism progressed. The high CV in subsequent minutes suggested individual differences in glucose metabolism.

Regarding the glucose variation across the three treatments, the results are presented as follows:

### Treatment 1 with 100 mg/kg

The variability analysis revealed a high CV in the glucose response, indicating that the 100 mg/kg dose of the extract did not regulate glucose homogeneously, as hyperglycemia remained elevated and variable.

### Treatment 2 with 150 mg/kg

EAAA at 150 mg/kg stabilized glucose more effectively compared to the 100 mg/kg dose, reducing hyperglycemia in a more homogeneous and controlled manner. This was attributed to a more consistent decrease in glucose levels, with low variability observed in post-administration values.

### Treatment 3 with 300 mg/kg

At this dose, basal glucose levels were the lowest, suggesting that the extract may have a long-term hypoglycemic effect. An extremely high CV% was observed in the first 30 minutes, indicating inconsistent initial effects of the 300 mg/kg dose across individuals. However, glucose levels progressively decreased by 90 minutes, leveling off and showing a sustained reduction. These values were the lowest among all groups, suggesting that this dose was the most effective for long-term glucose regulation.

Regarding the statistical tests to assess the presence of significant differences in glucose levels at 30, 60, and 90 minutes among the experimental groups (Control, 100 mg/kg, 150 mg/kg, and 300 mg/kg of AEAA), an analysis of variance (ANOVA) and the Waller-Duncan multiple range test were performed. The ANOVA in Table 6 showed significant differences among the treatments (p = 0.000), indicating that at least one group exhibited a distinct response in glucose reduction.

**Table 6.** ANOVA de la respuesta de glucosa en ratas tratadas con EAAA.

	Source of variation	Sum of squares	df	Mean square	F	Sig. (p-value)
**Mg/dL**	Entre grupos	37823.083	2	18911.542	8.524	0.000
	Dentro de grupos	206326.156	93	2218.561		
		244149.240	95			

The Duncan test (Alpha = 0.05) ( Table 7) was used to identify differences between groups. The results revealed that the 300 mg/kg dose showed a significant difference compared to the control group, indicating that this treatment at 90 minutes produced a significant reduction in blood glucose levels compared to untreated rats. At 60 minutes, the same treatment exhibited an intermediate effect but remained significantly different from the 30 minutes time point, which the least effective.

**Table 7.** Results of the duncan test for blood glucose levels at 30, 60 and 90 minutes.

Waller-Duncan			
Glucose	Sample size (N)	Subset for α = 0.05	
		1	2
90 min	32	139.41	
60 min	32	158.34	158.34
30 min	32		187.66

In contrast, the 100 mg/kg and 150 mg/kg doses showed no significant differences compared to the control group, suggesting they may not be sufficiently effective to impact blood glucose reduction. A practical interpretation de AEAA’s efficacy highlights the importance of higher doses (e.g., 300 mg/kg), which may be necessary to achieve a more consistent and effective effect.

A realistic interpretation of EAAA’s efficacy would suggest the importance of higher concentrations, which may be necessary to achieve a more consistent and effective therapeutic effect.

## 4. Discussion

Phytochemical analysis of *A. argomuelleri* leaves revealed the presence of flavonoids in moderate proportions, high levels of phenolic compounds, and the absence of sugars. This bioactives compounds, commonly found in other *Acalypha* species, may have been responsible for the observed effects on glucose regulation due to their highly significant hypoglycemic medicinal properties. Previous studies have documented that the hypoglycemic effects of plants associated with the presence of flavonoids and phenols, are often attributed to their ability to enhance the function of pancreatic tissue, specifically the β-cells. This effect may be achieved either by stimulating insulins secretion or by reducing intestinal glucose absorption. ^12, 29^

These results ( Table 1) suggest that, in the absence of AEAA, the glycemic homeostasis of the rats remained relatively stable over time, although individual fluctuations were observed. Such variations could be influenced by physiological factors such as basal metabolism, prior diet, or compensatory responses to fasting before treatment administration. ^30^

The results obtained with AEAA at a dose of 100 mg/kg showed a variable response in blood glucose levels in rats, indicating a partially regulatory effect with individual differences in response and, consequently, a lack of uniformity in outcomes. This variability could be related to the action of the extract´s bioactive compounds—mainly flavonoids and phenols—which are known to modulate glucose metabolism. Additionally, these variations might be attributed to genetic factors, differences in extract absorption, or the complexity of interactions involving flavonoids and phenols. ^31^

At a dose of 150 mg/kg, a progressive decrease in blood glucose levels was observed in most rats, reaching near-normal values at 60 and 90 minutes. The lower individual variability and grater stability in glucose levels at 90 minutes suggest that this dose of AEAA may be more effectively modulating glucose absorption and metabolism than the 100 mg/kg dose. This effect could be attributed to the action of bioactive compounds, primarily flavonoids and phenols, which according to various studies, can influence glycemic homeostasis through mechanisms such as the inhibition of digestive enzymes (α-glucosidase y la α-amylase) and stimulation of insulin secretion. ^32^ Furthermore, the observed response in this treatment indicates that, although the effect is significant, individual differences persist, which may be linked to genetic factors or variations in the absorption and bioavailability of the extract. ^33^

In contrast, the results obtained with AEAA at a dose of 300 mg/kg showed a faster, sustained, and more uniform reduction in blood glucose levels. The average basal glucose recorded before hyperglycemia induction was 78 mg/dL reaching a peak of 391 mg/dL, which is characteristic of the experimental model. However, by 90 minutes glucose values were significantly lower compared to other treatments, suggesting that this dose has a more pronounced and long-lasting hypoglycemic effect.

This more consistent response could be linked to a higher concentration of bioactive compounds that optimize glucose utilization in peripheral tissues, promoting more effective regulation of glycemic homeostasis. ^34^ Additionally, it has been proposed that flavonoids and phenols present in *Acalypha* species not only stimulate insulin secretion but also protect pancreatic β-cells by reducing oxidative stress and modulating the expression of pro- and anti-apoptotic genes. ^35, 36^

The 300 mg/kg dose demonstrated the highest hypoglycemic efficacy, with a sustained reduction in blood glucose levels to near-normoglycemia values at 90 minutes. This finding combined with the absence of reducing sugars in the extract, suggests that the optimal concentration of flavonoids (++) and phenols (+++) may act effectively through a multifactorial mechanism targeting multiple metabolic pathways: (1) inhibition of α-glucosidase, (2) modulation of intestinal glucose absorption, and (3) protection of pancreatic β-cells against oxidative stress — key mechanisms associated with antidiabetic effects in plants such as *M. alba* and other *Acalypha* species. ^8^

However, the variability observed (CV > 50% at 30 minutes) highlights the influence of individual factors such as basal metabolism, bioavailability of active compounds, and hormonal fluctuations, ^37^ which could affect treatment response and therapeutical efficacy. Future studies—including a comprehensive phytochemical analysis and chronic diabetes models—will clarify whether *A. argomuelleri* Briq. Possesses additional antidiabetic effects (e.g., improvement insulin resistance or reduction of systemic oxidative stress). Current evidence indicates that the hypoglycemic effect of AEAA is dose-dependent, suggesting that the highest dose is the most effective long-term. This finding aligns with other studies in experimental models, where doses ranging from 200 to 500 mg/kg showed significant glucose reduction by 120 minutes. ^38– 40^

Eight rats per group were used, which is an adequate and commonly accepted number in preclinical pharmacological evaluation studies. This sample size allowed statistically significant differences to be observed between groups, although it is recognized that further studies with larger samples could strengthen the evidence and facilitate the exploration of additional mechanisms.

While the results obtained provide evidence of the hypoglycemic effect of *A. argomuelleri* Briq., it is important to note that this study was conducted in an experimental model using healthy albino rats. Future studies could evaluate the extract´s impact on induced diabetes models to determine its effectiveness under pathological conditions. Additionally, more detailed phytochemical analyses would be relevant to identify the compounds responsible for the hypoglycemic effect and elucidate their mechanism of action.

Taken together, these findings suggest that AEAA could represent a natural alternative in complementary medicine for blood glucose control, with potential therapeutic applications in the management of type 2 diabetes mellitus (T2DM). However further studies are required to validate its efficacy in humans.

It is suggested that the findings could be a basis for studies in other animal models or in humans, although a direct clinical application is not yet proposed.

## 5. Conclusions

This study represents a pioneering contribution to the evaluation of the hypoglycemic effect of the aqueous extract of *Acalypha argomuelleri* Briq. (AEAA) in albino rats, as o prior documented research exists on the species in relation to blood glucose regulation. The key findings are as follows:

• The aqueous extract of *Acalypha argomuelleri* Briq. At a dose of 300 mg/kg, significantly reduced blood glucose levels in *Rattus rattus* var. *albinus* compared to the control group, demonstrating a dose-dependent hypoglycemic effect.
• The efficacy of the aqueous extract of *Acalypha argomuelleri* Briq. Increased with treatment duration, showing a progressive reduction in glucose levels due to its cumulative and sustained effect.
• The 90 minute treatment exhibited the greatest reduction in glucose levels, representing the optimal timepoint to maximize the hypoglycemic effect without inducing adverse effects.

## Ethical considerations

The corresponding permits were obtained from the National Forest and Wildlife Service (SERFOR) (Resolutions No. D000120-2023-MIDAGRI-SERFOR-ATFFS-CAJAMARCA and No. D000167-2024) for the collection of *Acalypha argomuelleri* Briq., as well as authorization from the Research Department of the Lambayeque Regional Hospital (HRL), including approval from the Institutional Research Ethics Committee for the use of animals (No. 026-2025).

## Data Availability

Zenodo. Hypoglycemic effect of the aqueous extract from Acalypha argomuelleri Briq. ‘Sweet stick’ leaves in Rattus rattus var. albinus, https://doi.org/10.5281/zenodo.16732470. ^41^ This project contains the following underlying data:
• TOLERANCE TEST DATABASE (Excel file).xlsx (Individual blood glucose data in rats by experimental group and day of evaluation). • ANOVA DATA SPSS.spv - (Results of the ANOVA analysis applying SPSS to the experimental data). •
Duncan_Acalypha_test (1).txt - (Results of the Duncan/Tukey post hoc test with detailed statistical significance by pairs). •
BASE DOF DATES TEST OF TOLERANCE.xlsx
•
MEDIA, DS Y CV ACALYPHA.docx
•
RESAULTS JANUARY 31.pdf The data is available under the terms of the Creative Commons Attribution 4.0 International licence (CC BY 4.0). Zenodo. Hypoglycemic effect of the aqueous extract from Acalypha argomuelleri Briq. ‘Sweet stick’ leaves in Rattus rattus var. albinus, https://doi.org/10.5281/zenodo.16732470. ^41^ This project contains the following underlying data: Author Checklist - Full 19_07_25.pdf The data is available under the terms of the Creative Commons Attribution 4.0 International licence (CC BY 4.0).
