# Supplementary material for: Hypoglycemic effect of the aqueous extract from Acalypha argomuelleri Briq. (Euphorbiaceae) 'Sweet stick' leaves in Rattus rattus var. albinus
Source: F1000Res. 2025 Oct 10;14:800. Originally published 2025 Aug 19. [Version 3] doi: 10.12688/f1000research.164673.3 (PMC12461185; doi:10.12688/f1000research.164673.3)
Supplement: Peer review history

# Reviewer report: Reviewer response for version 3

**DOI:** 10.5256/f1000research.188409.r422492

**Published:** 2025-10-14

**Authors:** Antonio Galán de Mera

**Affiliations:** 1 Universidad San Pablo-CEU, Madrid, Spain

As I indicated last time, my decision is ACCEPTED.

Is the work clearly and accurately presented and does it cite the current literature?

Yes

If applicable, is the statistical analysis and its interpretation appropriate?

Yes

Are all the source data underlying the results available to ensure full reproducibility?

Yes

Is the study design appropriate and is the work technically sound?

Yes

Are the conclusions drawn adequately supported by the results?

Yes

Are sufficient details of methods and analysis provided to allow replication by others?

Yes

Reviewer Expertise:

Plant systematics, Biogeography, Pharmacognosy.

I confirm that I have read this submission and believe that I have an appropriate level of expertise to confirm that it is of an acceptable scientific standard.

# Reviewer report: Reviewer response for version 2

**DOI:** 10.5256/f1000research.187885.r410404

**Published:** 2025-09-24

**Authors:** MARIO ANTONIO BOLARTE ARTEAGA, Bolarte Artega Mario Antonio

**Affiliations:** 1 UNIVERSIDAD CONTINENTAL, Lima, Peru; 2 Universidad Continental - Los Olivos Lima, Lima Region, Lima District, Peru

1. I **NTRODUCTION**

The introduction to the manuscript presents a clear and well-structured overview of the scientific and ethnobotanical context of Acalypha argomuelleri as a species of medicinal interest, citing other native Peruvian and foreign species. The authors successfully articulate the importance of preclinical studies in animal models with the need to scientifically validate the traditional uses reported in local communities. Likewise, an adequate literature review is included that places the work within the framework of current research on hypoglycaemic plants, allowing the reader to understand both the relevance from a biological point of view and the value from another point of view, which is the cultural value of the natural resource studied. The writing is fluid, coherent, and sufficient to justify the objective of the study, so I believe that this section does not require additional modifications. The manuscript addresses a relevant topic in the field of pharmacognosy and complementary medicine, evaluating the hypoglycaemic effect of Acalypha argomuelleri, a little-studied species with potential therapeutic application.

  

1. **METHODOLOGY**

 The methodology presented in the manuscript is clear and consistent with the objectives set out. The glucose tolerance test procedure, the administration of the extract in different doses, and the recording of the glycaemic response are described accurately, ensuring reproducibility in future studies. Likewise, the use of statistical analysis using ANOVA followed by Duncan's test is relevant and correctly applied, lending strength to the results. I believe that the methodology section does not require further modification, as it meets the standards of rigour and transparency expected in this type of research.

  

1. **RESULTS**

The results section is organised in a clear and coherent manner, adequately presenting both the experimental data and its statistical analysis. The tables accurately indicate the variations in glucose levels following the administration of the extract in different doses, which facilitates understanding of the findings. In addition, Duncan's test is correctly described, allowing significant differences between groups to be identified. The writing is objective and avoids premature interpretations, limiting itself to presenting the findings as they were obtained. I consider the results to be sufficient, well presented, and requiring no further modification.

  

1. **DISCUSSION**

The discussion is solid and adequately fulfils the purpose of interpreting the results in light of existing scientific evidence. The authors make relevant comparisons with other plants known for their hypoglycaemic activity, which places their findings in a broader context and gives the study international relevance. Furthermore, there is a balance between the interpretation of the data and the recognition of limitations, as well as the mention of future lines of research, demonstrating a forward-looking and responsible approach. The writing is clear, coherent and well structured, allowing the reader to understand both the scope and potential of the extract of the plant studied. I consider that this section does not require any modifications.

Is the work clearly and accurately presented and does it cite the current literature?

Yes

If applicable, is the statistical analysis and its interpretation appropriate?

Yes

Are all the source data underlying the results available to ensure full reproducibility?

Yes

Is the study design appropriate and is the work technically sound?

Yes

Are the conclusions drawn adequately supported by the results?

Yes

Are sufficient details of methods and analysis provided to allow replication by others?

Yes

Reviewer Expertise:

Pharmacogenomics, Medicina plants and Pharmacology

We confirm that we have read this submission and believe that we have an appropriate level of expertise to confirm that it is of an acceptable scientific standard.

# Response

**Published:** 2025-09-25

**Authors:** Jorge Guillermo Morales Ramos

**Affiliations:** Lambayeque, Universidad De San Martin de Porres - Filial Norte, Chiclayo, Lambayeque, Peru

We thank the reviewer for approving the article without comments.

# Reviewer report: Reviewer response for version 2

**DOI:** 10.5256/f1000research.187885.r414413

**Published:** 2025-09-16

**Authors:** Antonio Galán de Mera

**Affiliations:** 1 Universidad San Pablo-CEU, Madrid, Spain

I agree with this new amended version and recommend acceptance

 Thank you very much.

 Kind regards

Is the work clearly and accurately presented and does it cite the current literature?

Yes

If applicable, is the statistical analysis and its interpretation appropriate?

Yes

Are all the source data underlying the results available to ensure full reproducibility?

Yes

Is the study design appropriate and is the work technically sound?

Yes

Are the conclusions drawn adequately supported by the results?

Yes

Are sufficient details of methods and analysis provided to allow replication by others?

Yes

Reviewer Expertise:

Plant systematics, Biogeography, Pharmacognosy.

I confirm that I have read this submission and believe that I have an appropriate level of expertise to confirm that it is of an acceptable scientific standard.

# Reviewer report: Reviewer response for version 1

**DOI:** 10.5256/f1000research.181218.r407373

**Published:** 2025-09-08

**Authors:** Aku Enam Motto

**Affiliations:** 1 University of Lomé, Lomé, Togo

**RESEARCH ARTICLE**

**Hypoglycemic effect of the aqueous extract from Acalypha  argomuelleri Briq. 'Sweet stick' leaves in Rattus rattus var. albinus**

**Abstract**

 Introduction

 Reformulate it

**Material and methods**

 The authors should follow the structure for more clarification and understanding

**Animals**

**Plant collection and identification**

**Preparation of the aqueous extract**

**Phytochemical screening**

 This is a qualitative test, so there is no need to write +++ or ---

 Absence -  and Presence +

**Experimental design**

 The authors should mention the dose of glucose administered to the rats

 The authors did not mention whether the manipulation occurred in fasted rats or not

 Precision of the sex of the animals is also needed.

 The measurement of the glycemia must be extended in 2 hours ( 30 min, 60 min, 60 min; 120 and 180 min)

 For this kind of evaluation of the hypoglycemic activity, there are a lot of adequate protocols nowadays

 (refer to 1&2)

 For a better understanding, the authors must complete the test.

**Statistical analysis**

Is the work clearly and accurately presented and does it cite the current literature?

Partly

If applicable, is the statistical analysis and its interpretation appropriate?

Yes

Are all the source data underlying the results available to ensure full reproducibility?

No source data required

Is the study design appropriate and is the work technically sound?

Partly

Are the conclusions drawn adequately supported by the results?

Partly

Are sufficient details of methods and analysis provided to allow replication by others?

No

Reviewer Expertise:

Physiology, Pharmacology, toxicology

I confirm that I have read this submission and believe that I have an appropriate level of expertise to state that I do not consider it to be of an acceptable scientific standard, for reasons outlined above.

## References

[1] : Antihyperglycemic and Antioxidant Properties of Sida linifolia Juss. ex Cav. (Malvaceae) Hydroalcoholic Extract in ICR Mice. *Asian Science Bulletin* .2025;3(1) : 10.3923/asb.2025.08.19 8-19 10.3923/asb.2025.08.19

[2] : Potential Anti-hyperglycemic Activity of the Polyherbal Combination of Carica papaya L., Persea americana Mill., and Theobroma cacao L. Leaves Extract: In vitro, Ex vivo, In vivo Studies. *Journal of Advances in Medical and Pharmaceutical Sciences* .2025;27(2) : 10.9734/jamps/2025/v27i2751 54-66 10.9734/jamps/2025/v27i2751

# Response

**Published:** 2025-09-21

**Authors:** Jorge Guillermo Morales Ramos

**Affiliations:** Lambayeque, Universidad De San Martin de Porres - Filial Norte, Chiclayo, Lambayeque, Peru

Abstract

 The sex of the rats was added to the summary.

 Introduction

 Reformulate

 It has been reformulated by adding Peruvian plants with hypoglycaemic effects (page 3, paragraph 10), article v2.

 Materials and methods

 Authors should follow the structure for greater clarity and understanding.

 Animals

 The reviewer's suggestion was accepted, incorporating the sex of the study animals and indicating that they were male.

 Plant collection and identification

 The methods of Cerrate and Romagosa et al. were used for the collection of botanical material.

  Cerrate E. Manera de preparar plantas para un herbario. Lima: Editorial UNMSM; 1969.

 Romagosa J, Rosales S, Rupérez RF, Crespo R. Enciclopedia de medicina naturalista y alternativa; 2000.

 Preparation of the aqueous extract

 Phytochemical screening

 The methodology mentioned the method followed by García Granados (paragraph 7, p.3, ref. 27), which describes the stages of the process.

 This is a qualitative test, so it is not necessary to write +++ or ---.

 Absence - and Presence +

 Version 2 of the article states that the test was qualitative with semi-quantitative estimation (p. 3, paragraph 1, Phytochemical analysis, ref. 28).

 Experimental design

 The authors should mention the dose of glucose administered to the rats.

 Paragraph 2 of the experimental design section indicates that the dose of glucose administered was 5 ml.

 The authors did not mention whether the manipulation was performed on fasted rats or not.

 The experimental design section mentions the conditions referred to by the reviewer (p. 7, paragraph 2).

 It is also necessary to specify the sex of the animals.

 We appreciate the reviewer's suggestion to include the sex of the rats, which were males paragraph 2(a).

 Blood glucose measurement should be extended to 2 hours (30 min, 60 min, 60 min; 120 and 180 min).

 We appreciate the reviewer's suggestion regarding the inclusion of additional times (120 and 180 minutes). In our study, 30, 60, and 90 minutes were selected because these intervals are widely used in glucose tolerance tests in preclinical models with plant extracts, as they allow both the post-load peak and the early phase of blood glucose return to be captured (Tripathi et al., 2014; Narmadha, et al., 2017). With this design, it was possible to identify the maximum hypoglycaemic effect of the extract in the critical window where changes are most evident. While we recognise that extended measurements up to 120 or 180 minutes may provide additional information, the main objective of this exploratory study was to evaluate the early hypoglycaemic response, which is adequately reflected in the 90 minutes. Undoubtedly, future studies —especially in diabetic models and chronic evaluations— will include longer times.

 Tripathi AK, Kohli S. Pharmacognostical standardization and antidiabetic activity of Syzygium cumini (Linn.) barks (Myrtaceae) on streptozotocin-induced diabetic rats. J Complement Integr Med. 2014 Jun;11(2):71-81. doi: 10.1515/jcim-2014-0011. PMID: 24760764.

 Narmadha R, Devaki K. Toxicological evaluation and oral glucose tolerance test of ethanolic leaf extract of Barleria cristata L. in wistar albino rats. Int J Basic Clin Pharmacol [Internet]. 2017 Feb. 2 [cited 2025 Sep. 12];2(6):742-6. Available from: https://www.ijbcp.com/index.php/ijbcp/article/view/1359

# Reviewer report: Reviewer response for version 1

**DOI:** 10.5256/f1000research.181218.r410405

**Published:** 2025-09-03

**Authors:** Antonio Galán de Mera

**Affiliations:** 1 Universidad San Pablo-CEU, Madrid, Spain

I think this is a very interesting type of manuscript that scientifically demonstrates the traditional use of certain plants in Peru that could be adopted by occidental medicine. Perhaps it would be useful to indicate whether the extracts could have secondary effects (although this is not the aim of this work) and what the subsequent steps would be in order to develop a pharmaceutical preparation.

 Although it is a pharmacological manuscript, the botanical aspects of the plant should be dealt with in more detail. For example, the way in which its ecology is dealt with is very superficial, when it is precisely a natural resource that could lead to the establishment of a nature reserve.

**Other points:**

 In the title, add the family after *Acalypha argomuelleri* Briq. **(Euphorbiaceae).**

 The caption for **Table 6** should be in English.

**In paragraph 6 of the introduction, **I believe that if the new plant that causes hypoglycaemia is Peruvian, more plants from Peru that cause this effect should be added, and not just *Geranium ayavacense. *Furthermore, three paragraphs below, a very similar paragraph is repeated, and what is detailed in it should be included here. Join two paragraphs.

 In paragraph 7, line 4: THESE ARE NOT SYSTEMATIC REVIEWS OF PLANTS (BOTANICAL EXPRESSION), THEY ARE PHYTOCHEMICAL AND PHARMACOLOGICAL REVIEWS: It should be stated: as synthesised in our ongoing phytochemical and pharmacological reviews.

**Paragraph 12 and 13 of the introduction:**

**'the humid tropical biome': **This is taken from IPNI, but IPNI is not accurate for ecological data. What is its distribution in Peru? Add some bibliographical references about its distribution. There is no humid tropical biome. It should be indicated whether it is a humid tropical forest, humid tropical shrubs, a savannah-type forest, etc., and also the bibliographical reference from which this information is taken. IF WE ARE DISCUSSING ABOUT A NATURAL RESOURCE, IT IS VERY IMPORTANT TO CORRECTLY INDICATE THE ECOLOGICAL CONDITIONS OF THE LOCATION. A PHARMACOLOGICAL STUDY CAN LEAD TO A WHOLE CONSERVATION STRATEGY.

**'traditional use in folk medicine': **What is it used for in traditional medicine? Please specify it, and add a bibliographical reference.

**Methods, paragraph 5: **WHY HAVE ONLY THE LEAVES BEEN USED? AND WHY HAVE THEY BEEN COLLECTED WHEN THE PLANT IS IN BLOOM? PERHAPS BECAUSE THAT IS HOW IT IS DONE IN TRADITIONAL MEDICINE? THE REASON SHOULD BE STATED.

**In that paragraph, after Peru: **Cajamarca Department, Peru. Also add coordinates and date of collection. National University of Trujillo: Is the registered name IN ENGLISH? OR IS IT  UNIVERSIDAD NACIONAL DE TRUJILLO?

 After registration, the voucher number must be added to the HUT herbarium, which is the acronym for the herbarium.

 Discussion: Phytochemical analysis: The intensity of the color reaction qualitatively indicated...but also quantitative? I believe you are showing colour intensity according to quantity, so wouldn't that also be quantitative analysis? Furthermore, in paragraph 2 of the **results**, the authors wrote the sentence ‘Qualitative phytochemical analysis revealed an abundant presence of phenols, a moderate amount of flavonoids and diterpenes, and a small **quantity **of cardiotonic glycosides’.

**Discussion, paragraph 1: **The authors wrote: 'Previous studies have documented that the hypoglycemic effects of plants associated' **Which plants?**

 Paragraph 10 of the discussion states: ' *Taken together, these findings suggest that AEAA could represent a natural alternative in complementary medicine for blood glucose control, with potential therapeutic applications in the management of type 2 diabetes mellitus (T2DM).*

*However further studies are required to validate its efficacy in humans.' *What success rate could extracts from this plant have in humans, considering that it is used in traditional medicine? Could a medicine be manufactured that could be dispensed in pharmacies? These would be interesting points to add to the discussion.

Is the work clearly and accurately presented and does it cite the current literature?

Yes

If applicable, is the statistical analysis and its interpretation appropriate?

Yes

Are all the source data underlying the results available to ensure full reproducibility?

Yes

Is the study design appropriate and is the work technically sound?

Yes

Are the conclusions drawn adequately supported by the results?

Yes

Are sufficient details of methods and analysis provided to allow replication by others?

Yes

Reviewer Expertise:

Plant systematics, Biogeography, Pharmacognosy.

I confirm that I have read this submission and believe that I have an appropriate level of expertise to confirm that it is of an acceptable scientific standard.

# Response

**Published:** 2025-09-08

**Authors:** Jorge Guillermo Morales Ramos

**Affiliations:** Lambayeque, Universidad De San Martin de Porres - Filial Norte, Chiclayo, Lambayeque, Peru

1. In the title, add the family after *Acalypha argomuelleri* Briq. **(Euphorbiaceae).**

 Answer: The addition suggested by the reviewer was made.

 2. The caption for **Table 6** should be in English.

 Answer: The legend for Table 6 was translated.

**3. In paragraph 6 of the introduction, **I believe that if the new plant that causes hypoglycaemia is Peruvian, more plants from Peru that cause this effect should be added, and not just *Geranium ayavacense. *Furthermore, three paragraphs below, a very similar paragraph is repeated, and what is detailed in it should be included here. Join two paragraphs.

 Answer:The suggested paragraphs were merged, mentioning some plants of Peruvian origin that were already mentioned in the article.

 4. In paragraph 7, line 4: THESE ARE NOT SYSTEMATIC REVIEWS OF PLANTS (BOTANICAL EXPRESSION), THEY ARE PHYTOCHEMICAL AND PHARMACOLOGICAL REVIEWS: It should be stated: as synthesised in our ongoing phytochemical and pharmacological reviews.

 Answer: The reviewer's suggestion has been added to the text.

**5. Paragraph 12 and 13 of the introduction:**

**'the humid tropical biome': **This is taken from IPNI, but IPNI is not accurate for ecological data. What is its distribution in Peru? Add some bibliographical references about its distribution. There is no humid tropical biome. It should be indicated whether it is a humid tropical forest, humid tropical shrubs, a savannah-type forest, etc., and also the bibliographical reference from which this information is taken. IF WE ARE DISCUSSING ABOUT A NATURAL RESOURCE, IT IS VERY IMPORTANT TO CORRECTLY INDICATE THE ECOLOGICAL CONDITIONS OF THE LOCATION. A PHARMACOLOGICAL STUDY CAN LEAD TO A WHOLE CONSERVATION STRATEGY.

 Answer: The additions suggested by the reviewer were made, indicating the ecology of the plant, its use and traditional preparation.

**6. 'traditional use in folk medicine': **What is it used for in traditional medicine? Please specify it, and add a bibliographical reference.

 Answer: The reviewer's suggestion was accepted and the reference was added.

**7. Methods, paragraph 5: **WHY HAVE ONLY THE LEAVES BEEN USED? AND WHY HAVE THEY BEEN COLLECTED WHEN THE PLANT IS IN BLOOM? PERHAPS BECAUSE THAT IS HOW IT IS DONE IN TRADITIONAL MEDICINE? THE REASON SHOULD BE STATED.

**In that paragraph, after Peru: **Cajamarca Department, Peru. Also add coordinates and date of collection. National University of Trujillo: Is the registered name IN ENGLISH? OR IS IT  UNIVERSIDAD NACIONAL DE TRUJILLO?

 After registration, the voucher number must be added to the HUT herbarium, which is the acronym for the herbarium.

 Answer: The relevant corrections were made, adding the use and preparation of the plant, as well as an explanation of how the leaves were used in the experiment. Coordinates and the date of collection were also added, according to the herbarium voucher.

 Discussion: 

 8. Phytochemical analysis: The intensity of the color reaction qualitatively indicated...but also quantitative? I believe you are showing colour intensity according to quantity, so wouldn't that also be quantitative analysis? Furthermore, in paragraph 2 of the **results**, the authors wrote the sentence ‘Qualitative phytochemical analysis revealed an abundant presence of phenols, a moderate amount of flavonoids and diterpenes, and a small **quantity **of cardiotonic glycosides’.

 Answer: The suggestion to replace qualitative phytochemical analysis with semi-quantitative phytochemical analysis is accepted.

**Discussion, paragraph 1: **The authors wrote: 'Previous studies have documented that the hypoglycemic effects of plants associated' **Which plants?**

 Paragraph 10 of the discussion states: ' *Taken together, these findings suggest that AEAA could represent a natural alternative in complementary medicine for blood glucose control, with potential therapeutic applications in the management of type 2 diabetes mellitus (T2DM).*

*However further studies are required to validate its efficacy in humans.' *What success rate could extracts from this plant have in humans, considering that it is used in traditional medicine? Could a medicine be manufactured that could be dispensed in pharmacies? These would be interesting points to add to the discussion.

 Answer: We thank the reviewer for the clarification and suggestion regarding the structure of the paragraph, and the relevant changes have been made in the paragraphs 1 y 10.
